# Investigating the anatomical relationship between the maxillary molars and the sinus floor in a Chinese population using cone-beam computed tomography

**DOI:** 10.1186/s12903-019-0969-0

**Published:** 2019-12-16

**Authors:** Xi Zhang, Yan Li, Yi Zhang, Fengling Hu, Bin Xu, Xiaojun Shi, Liang Song

**Affiliations:** 1 Department of Stomatology, Shanghai Fifth People’s Hospital, Fudan University, Shanghai, 200240 China; 2 Department of Health Management, The Third Xiangya Hospital, Central South University, Changsha, 410013 China; 3 Radiology Department, Shanghai Prison General Hospital, Shanghai, 201318 China

**Keywords:** Cone beam computed tomography, Maxillary molar, Maxillary sinus

## Abstract

**Background:**

The anatomical relationship between the root apices of maxillary molars and the maxillary sinus floor (MSF) is important for the treatment of dental implantations and endodontic procedures. In this study, the detailed anatomical relationships between the root apices of maxillary molars and the MSF were studied in a Chinese population using CBCT.

**Methods:**

We collected the CBCT data files of patients who visited the stomatology outpatient clinic in Shanghai Fifth People’s Hospital, Fudan University from January 1, 2017 to January 1, 2019 and measured the following items: the distance between the molar root apices and the MSF, the thickness of the mucosa and cortical bone of the MSF closest to the root apices, and the angle between the buccal and palatal roots.

**Results:**

The shortest distances between the root apices and the MSF were 1.57 ± 3.33 mm (the mesiobuccal root of the left second molar) and 1.61 ± 3.37 mm (the mesiobuccal root of the right second molar). Apical protrusion over the inferior wall of the sinus most often occurred in the mesiobuccal root of left second molar (frequency, 20.5%). The mucosa of the MSF was thinnest at the distobuccal root of the right second molar (1.52 ± 0.85 mm), the cortical bone of the MSF was thinnest at the mesiobuccal root of the right second molar (0.46 ± 0.28 mm), and the angle between the buccal and palatal roots ranged from 12.01° to 124.2° (42.36 ± 24.33 °).

**Conclusions:**

Among the root apices of the maxillary molars, the mesiobuccal root apex of the left second molar was closest to the MSF, and it had the highest incidence of protrusion into the sinus. The unique anatomical relationship between the maxillary molars and the MSF in this Chinese population is critical for treatment planning for dental implantation or endodontic procedures.

## Background

The anatomical relationship between the root apex of the maxillary molars and the inferior wall of the maxillary sinus (i.e., the maxillary sinus floor, MSF) is critical for planning dental implantation, tooth extraction, and endodontic procedure s[1–4]. The vertical relationship between the MSF and the maxillary root apices varies according to age and the size and degree of pneumatization of the maxillary sinu s[5]. Occasionally, there is only one layer of mucous or cortical bone in the MSF, and this increases the risk of oroantral fistula or infection in the maxillary sinus. Therefore, identification of the degree of proximity and the thickness of the mucosa and cortical bone between the root apex and the MSF is critical for surgical procedure s[6, 7].

Conventional radiographic exams, including periapical and panoramic radiographs, are commonly used for the study of the anatomical relationship between molar root apices and the MS F[8]. However, these two-dimensional images have limitations that may prevent the correct interpretation of the relationship between periapical lesions and the MS F[8, 9]. Cone beam computed tomography (CBCT) is a three-dimensional imaging method that has been used for craniofacial radiology. Further, CBCT offers multi-planar views and overcomes the limitations associated with two-dimensional imaging, such as distortion, magnification, and superimpositio n[3, 10].

The anatomy of the midface, including the maxillary sinus, differs significantly among the human specie s[11]. Therefore, it is reasonable to postulate that the anatomical proximity between the molar root apex and the MSF varies between populations. Although many studies have focused on the vertical relationship between the maxillary molar root apices and the MSF in Brazilians, Russian, Korean and Turkey,[7, 12–14] detailed anatomical studies of the proximity of molar root apices and the MSF in the Chinese population are rar e[15, 16].

Therefore, this study aimed to evaluate the anatomical relationship between the maxillary molar root apices and the MSF in a Chinese population using CBCT, by measuring the vertical relationships between the MSF and the roots of the molars, distance between the molar root apices and the MSF, thickness of the mucosa and cortical bone of the MSF closest to the root apices, and angle between the buccal and palatal roots.

## Methods

### Samples

This research was approved by the Ethics Committee of Shanghai Fifth People’s Hospital, Fudan University (protocol #2016023) in 2016. Written informed consent to participate was obtained from all of the participants. The CBCT data was collected from 200 patients (96 male and 104 female; mean age, 34 years; range, 18–50 years) who visited the out-patient clinic of stomatology. The sample consisted of 800 maxillary first and second molars.

Patients were excluded from participation if one maxillary molar was missing from either side of the mouth (excluding the third molars) or if they had a history of orthodontic treatment, root canal therapy for the posterior teeth, significant periodontal disease/bone loss, fused roots or less or more than 3 roots, systematic bone disease, or a tumor involving the maxillary bone.

### Image collection and measurements

CBCT images were obtained with the Planmeca ProMax® 3D Max (Planmeca, Finland). The technical data were as follows: anode voltage, 90 kV; anode current, 12 mA; and scan time, 27 s. The voxel sizes used for reconstruction were 80 μm, and the multi-planar views were completed with Romexis software (Planmeca Romexis®) by one oral radiologist. The sections for measuring were selected when the tip of the root was presented in the coronal or sagittal plane.

**Fig. 1:**
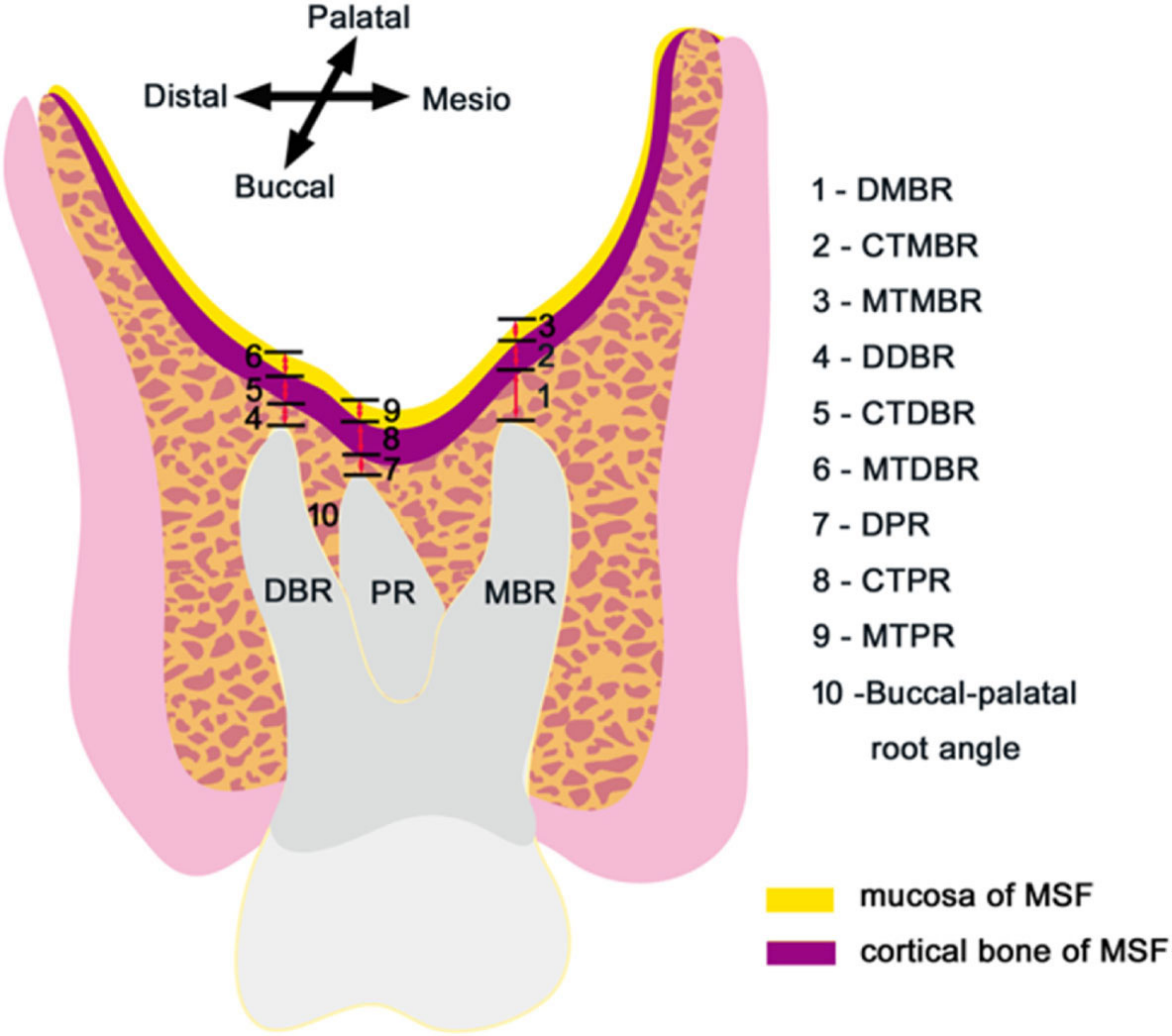
Measurements of maxillary molars using CBCT cross-sectional images. The measurements include 1-the distance between the mesiobuccal root apex and the inferior wall of the MSF (DMBR), 2-the cortical thickness of the MSF closest to the mesiobuccal root apex (CTMBR), 3-the mucosa thickness of the MSF closest to the mesiobuccal root apex (MTMBR), 4-the distance between the distobuccal root apex and the inferior wall of the MSF (DDBR), 5-the cortical thickness of the MSF closest to the distobuccal root apex (CTDBR), 6-the mucosa thickness of the MSF closest to the distobuccal root apex (MTDBR), 7-the distance between the palatal root apex and the inferior wall of the MSF (DPR), 8-the cortical thickness of the MSF closest to the palatal root apex (CTPR), 9-the mucosa thickness of the MSF closest to the palatal root apex (MTPR), and 10-the angle between the buccal and palatal roots

The measurements included the distance between the root apices of the maxillary molars and the MSF, the thickness of the mucosa and cortical bone of the MSF at the root apices, and the angle between the buccal and palatal roots. The detailed items included the distance between the mesiobuccal root apex and the inferior wall of the MSF (DMBR), the distance between the distobuccal root apex and the inferior wall of the MSF (DDBR), the distance between the palatal root apex and the inferior wall of the MSF (DPR), the cortical thickness of the MSF closest to the mesiobuccal root apex (CTMBR), the cortical thickness of the MSF closest to the distobuccal root apex (CTDBR), the cortical thickness of the MSF closest to the palatal root apex (CTPR), the mucosa thickness of the MSF closest to the mesiobuccal root apex (MTMBR), the mucosa thickness of the MSF closest to the distobuccal root apex (MTDBR), and the mucosa thickness of the MSF closest to the palatal root apex (MTPR) (Fig. 1).

**Fig. 2:**
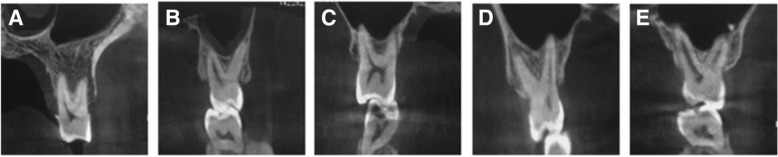
Different types of vertical relationships between the MSF and the root apices of the maxillary molars. **a.** Type I: the MSF is located above the connection between the buccal and palatal root apices, **b.** Type II: the MSF is located below the connection between the buccal and palatal root apices (without an apical protrusion over the MSF), **c.** Type III: an apical protrusion is observed over the MSF at the buccal root apex, **d.** Type IV: an apical protrusion is observed over the MSF at the palatal root apex, and **e.** Type V: apical protrusions are observed over the MSF at the buccal and palatal root apices

CBCT cross-sectional images were used to evaluate the vertical relationships between the root apices of the maxillary molars and the MSF, and these relationships were classified into five categories (Type 1–5) according to the following criteria described by Kwak et al .[12]: Type I, the MSF is located above the connection between the buccal and palatal root apices; Type II, the MSF is located below the connection between the buccal and palatal root apices, without an apical protrusion over the MSF; Type III, an apical protrusion is observed over the MSF at the buccal root apex; Type IV, an apical protrusion is observed over the MSF at the palatal root apex; and Type V, apical protrusions are observed over the MSF at the buccal and palatal root apices (Fig. 2a-e). The angle between the lines from the upper tangent point of the curvature of furcation to the point of the end tangent point of each root apex was calculated.

#### Statistical analysis

Statistical analysis was performed using SPSS 17.0. Data are presented as the mean ± standard deviation (SD). Unpaired t-tests were used to test whether there was any difference between men and women, and paired t-tests were used to test whether there was any difference between left and right sites. For all analyses, *p* < 0.05 was considered statistically significant.

## Results

### Comparison of sex and location on the relationship between the maxillary molars and the MSF

We evaluated the influence of sex (male vs female) and location (left vs right side) on the anatomical relationship between the maxillary molars and maxillary sinuses in 104 women and 96 men. Our results demonstrated that the distance between the root apex of the maxillary molars and the MSF, cortical bone thickness of the MSF, mucosal thickness of the MSF, maxillary molar root angle, the rates of different types of relationships (Type 1–5) between the maxillary molar roots and the MSF, and the proportion of maxillary sinus penetration were not significantly different between men and women or the left and right sides (*P >* 0.05).

### The distance between the root apices of the maxillary molars and the MSF

We compared the distances between the root apices of the maxillary molars and the MSF. The shortest distances between the root apices and the MSF were 1.57 ± 3.33 mm (the mesiobuccal root of the left second molar) and 1.61 ± 3.37 mm (the mesiobuccal root of the right second molar) (Table 1). Among the maxillary molar roots, protrusion over the MSF most commonly occurred in the mesiobuccal root of the left second molar (frequency, 20.5%) (Table 2).

**Table 1:** Distance between the root apex of the maxillary molars and the MSF

tooth position	n	MBR	DBR	PR
16	200	2.17 ± 3.44	2.36 ± 3.23	2.62 ± 3.52
17	200	1.61 ± 3.37	1.95 ± 3.44	2.91 ± 3.07
26	200	2.31 ± 3.10	2.49 ± 3.47	2.33 ± 3.20
27	200	1.57 ± 3.33	1.90 ± 3.17	2.55 ± 3.10

Tooth position 16 represents the right first molar, 17 represents the right second molar, 26 represents left first molar, and 27 represents left second molar

*MBR*, the distance between the apex of the mesiobuccal root and the inferior wall of the *MSF*; *DBR*, the distance between the apex of the distobuccal root and the inferior wall of the *MSF*; *PR*, the distance between the apex of the palatal root and the inferior wall of the *MSF*. Unit: mm; data are presented as the mean *SD*

**Table 2:** Proportion of apical protrusion of the maxillary molars

tooth position	MBR	DBR	PR	Whole tooth
16	26 (13%)	17 (8.5%)	31 (15.5%)	47 (23.5%)
17	32 (16%)	25 (12.5%)	10 (5%)	50 (25%)
26	16 (8%)	17 (8.5%)	27 (13.5%)	41 (20.5%)
27	41 (20.5%)	31 (15.5%)	14 (7%)	62 (31%)

Tooth position 16 represents the right first molar, 17 represents the right second molar, 26 represents left first molar, and 27 represents left second molar

*MBR*, mesiobuccal root; *DBR*, distobuccal root, *PR*: the palatal root. Whole tooth, at least one root protruded into the maxillary sinus

### The thickness of the cortical bone and mucosa of the MSF closest to the root apices

We evaluated the thickness of the cortical bone and mucosa of the MSF closest to the mesiobuccal, distobuccal, and palatal root apices and compared these thicknesses. Our results revealed that the cortical bone of the MSF was thinnest at the mesiobuccal root apex of the right second molar (0.45 ± 0.28 mm) and thickest at the palatal root apex of the left first molar (0.52 ± 0.39 mm) (Table 3).

**Table 3:** Cortical thickness of the *MSF* at the root apices of the maxillary molars

tooth position	n	CTMBR	CTDBR	CTPR
16	200	0.50 ± 0.30	0.49 ± 0.27	0.50 ± 0.31
17	200	0.45 ± 0.28	0.48 ± 0.34	0.50 ± 0.46
26	200	0.51 ± 0.49	0.50 ± 0.43	0.52 ± 0.39
27	200	0.49 ± 0.29	0.50 ± 0.67	0.47 ± 0.35

Tooth position 16 represents the right first molar, 17 represents the right second molar, 26 represents left first molar, and 27 represents left second molar. *CTMBR*, cortical thickness of the *MSF* at the mesiobuccal root apex; *CTMDR*, cortical thickness of the *MSF* at the distobuccal root apex; *CTPR*, cortical thickness of the *MSF* at the palatal root apex. *n* teeth number; unit: mm; data are presented as the mean *SD*

The mucosa of the MSF was thinnest at the distobuccal root of the right second molar (1.52 ± 0.85 mm) and thickest at the distobuccal root apex of the left first molar (1.96 ± 1.03 mm) (Table 4).

**Table 4:** The mucosal thickness of the *MSF* at the root apices of the maxillary molars

tooth position	n	MTMBR	MTDBR	MTPR
16	200	1.77 ± 0.86	1.83 ± 1.03	1.83 ± 1.17
17	200	1.66 ± 1.00	1.52 ± 0.85	1.70 ± 0.65
26	200	1.94 ± 0.98	1.96 ± 1.03	1.90 ± 1.12
27	200	1.78 ± 0.72	1.72 ± 1.05	1.77 ± 1.19

Tooth position 16 represents the right first molar, 17 represents the right second molar, 26 represents left first molar, and 27 represents left second molar. *MTMBR*, mucosal thickness of the *MSF* at the mesiobuccal root apex; *MTDBR*, mucosal thickness of the *MSF* at the distobuccal root apex; *MTPR*, mucosal thickness of the *MSF* at the palatal root apex. *n* teeth number; unit: mm; data are presented as the mean *SD*

### The rates of different types of relationships (type 1–5) between the maxillary molar roots and the MSF

The rates of the different types of relationships (Type 1–5) between the maxillary molar roots and the MSF are shown in Table 5. The results demonstrated that Type 1 relationships were most commonly observed in all of the maxillary molars (52.5–62.0%); Type IV relationships were most uncommonly observed in the right second molar (1.5%), left first molar (6.0%), and left second molar (2.0%); and Type V relationships were most uncommonly observed in the right second molar (3.5%) (Table 5).

**Table 5:** Types of relationships between the maxillary molar roots and the maxillary sinus floor

tooth position	n	Type I	Type II	Type III	Type IV	Type V
16	200	105 (52.5%)	47 (23.5%)	18 (9%)	17 (8.5%)	13 (6.5%)
17	200	120 (60%)	43 (21.5%)	27 (13.5%)	3 (1.5%)	7 (3.5%)
26	200	108 (54%)	56 (28%)	12 (6%)	12 (6%)	15 (7.5%)
27	200	124 (62%)	28 (14%)	32 (16%)	4 (2%)	10 (5%)
sum	800	457 (57.125%)	174 (21.75%)	89 (11.13%)	36 (4.5%)	45 (5.6%)

Tooth position 16 represents the right first molar, 17 represents the right second molar, 26 represents left first molar, and 27 represents left second molar

### The angle between the buccal and palatal roots

The angles between the buccal and palatal roots in different molars are shown in Table 6. The smallest angle was observed in the left second molar of Type V relationships (31.55 ± 26.03°), and the largest angle was observed in the right first molar of Type I relationships (56.47 ± 29.30°) (Table 6). The angles between the buccal and palatal roots in Type I were significantly larger than that in the other types (*P* < 0.05, one-way ANOVA). There were no significant differences among Type II, Type III, Type IV, and Type V (*P* > 0.05, one-way ANOVA).

**Table 6:** The angle between the buccal and palatal roots of the maxillary molars

tooth position	n	Type I	Type II	Type III	Type IV	Type V
16	200	56.47 ± 29.30	37.71 ± 16.67	37.73 ± 22.74	36.40 ± 19.04	36.26 ± 20.13
17	200	41.84 ± 25.88	35.58 ± 17.55	36.77 ± 17.30	35.98 ± 7.17	34.60 ± 21.52
26	200	52.88 ± 28.47	38.89 ± 28.47	35.68 ± 17.03	37.29 ± 19.74	38.33 ± 19.31
27	200	40.99 ± 24.28	35.69 ± 17.70	36.09 ± 14.83	37.56 ± 11.86	31.55 ± 26.03

Tooth position 16 represents the right first molar, 17 represents the right second molar, 26 represents left first molar, and 27 represents left second molar. Unit:°; data are presented as the mean *SD*

## Discussion

This study aimed to evaluate the anatomical relationship between the first and second maxillary molars and the maxillary sinus and provide a basis for oral clinical treatment.

The maxillary sinus is filled with liquid at birth. During growth and development, the liquid is gradually absorbed and gas begins to fill the sinus cavity. This process is called maxillary sinus gasificatio n[17]. Studies have shown that maxillary sinus gasification is generally completed at the age of 18 year s[5]. In order to avoid the influence of maxillary sinus gasification and guide adult clinical operations, CBCT images of the completed maxillary sinus gasification were analyzed in this study.

The anatomical relationships between the first and second molars (800 teeth) and the maxillary sinus were divided into five types (Type 1–5). Type I relationships were most commonly observed (57.125%), and 21.75, 11.13, 4.5, and 5.6% were Type II, Type III, Type IV, and Type V relationships, respectively. We compared the anatomical differences between different ethnicities. Estrela et al .[7] studied the anatomic relationship between the maxillary molars and the maxillary sinus in Brazil and reported that Type II relationships were most commonly observed (Table 7). These differences indicate that ethnicity may impact the anatomical relationship between the first or second molars and the maxillary sinus. Yurdabakan et al .[14] studied the anatomic relationship between the maxillary third molars and the maxillary sinus in Turkey and found that the most common vertical relationship was that the teeth roots were not contacting with the sinus floor, which is the same as the maxillary first and second molars in our study (Table 7).

**Table 7:** Comparison of current study with other studies

Project	Object	Sample number	Vertical relationship between the roots of the maxillary molar and the MSF	Distance between the root apices and the MSF	MSF thickness of the cortical bone closest to root apices	MSF thickness of the mucosa closest to root apices	Apical protrusion over the inferior wall of the sinus	The angle between the buccal and palatal roots of the maxillary molar
Current study	Chinese populaion	200 patients	The most common relationship was the MSF above the connection of the buccal and palatal root apices for first and second molar.	The shortest were observed at the mesiobuccal root of the left second molar, 1.57 ± 3.33 mm.	The thinnest were observed at the mesiobuccal root of the right second molar, 0.46 ± 0.28 mm.	The thinnest were observed at the distobuccal root of the right second molar,1.52 ± 0.85 mm.	It was most often occurred in the mesiobuccal root of left second molar (frequency, 20.5%).	The largest was observed in the right first molar (56.47 ± 29.30) when the MSF is located above the connection between the buccal and palatal root apices.
Estrela et al .[7]	Brazilian population	202 patients	The most common relationship was the MSF located below the level connecting the buccal and palatal root apices without an apical protrusion over the MSF for first and second molar.	The shortest were observed at the mesiobuccal root of the second molar, 0.36 ± 1.17 mm.	The thinnest were observed at the mesiobuccal root of the left second molar, 0.65 ± 0.41 mm.	NA	It was most often occurred in the second molar (frequency, 40%).	NA
Razumova et al .[13]	Russian population	325 patients	The most common relationship was MSF located below the level connecting the buccal and palatal root apices without an apical protrusion over the MSF for first and second	NA	The thinnest were observed at the mesiobuccal root of the second molar, 0.54 ± 1.19 mm.	NA	NA	NA
Yurdabakan et al .[14]	Turkish population	394 patients	The most common relationship was the MSF above the connection of the buccal and palatal root apices for the third molar.	NA	NA	NA	The frequency of maxillary third molar protusion into the MSF was 40.4%.	NA

Gu et al .[18] also studied the relationship between the maxillary sinus and the maxillary molars in China. But they only divided the relationship between the maxillary posterior teeth and the MSF into three types: Type OS (the root apex extendingbelow/outside the MSF), Type CO (the root apex contacting with the MSF), Type IS (the root apex extendingabove/inside the MSF). And they mostly focused on the impact of age and the absence of adjacent teeth on the relationship. The research from Ananda et al .[19] in China used another way to study the relationship between the maxillary sinus and the first maxillary molar. They divided the anteroposterior relationship into three categories: Type I (anterior to the mesiobuccal root of the maxillary first molar), Type II (posterior to the distobuccal root of the maxillary first molar), Type III (Anterior wall of the maxillary sinus was between the mesiobuccal and distobuccal roots of the maxillary first molar). Different from them our five type division can show more information.

The left second maxillary molar (31%) had the highest rate of protrusion into the MSF, and the shortest distance (1.57 ± 3.33 mm) from the root apex to the MSF (i.e., the distance from the mesiobuccal root to the MSF). Estrela et al .[7] also evaluated the relationship between the MSF and the root apices of the maxillary posterior teeth. Similar to our findings, Estrela et al .[7] reported that the shortest distance from the root apices to the MSF was the distance from the mesiobuccal root of the second molar to the MSF (Table 7).

The study by Lu et al .[20] in China evaluated maxillary sinus mucosal thickening affected by periodontitis of the maxillary premolars/molars and found that the maxillary sinus mucosal thickening increased dramatically as the severity of apical periodontitis increased. Different from them, we measured the thickness of the cortical bone and mucosa of the MSF in order to understand the impact of the relationship between the maxillary molar root and the maxillary sinus on these structures in healthy condition. The cortical thickness of the MSF was 0.45 ± 0.28 mm at the mesiobuccal root of the right second molar in the current study. When cortical thickness is decreased, it is more easily damaged by periapical periodontitis, and inflammation may spread to the MS F[21]. Further, thinner cortical bones are less likely to achieve primary stability of implants than thicker cortical bones after maxillary sinus augmentatio n[22, 23]. According to our study, the mucosal thickness of the MSF at the root apices of the maxillary molars was around 2 mm, and the mucosa was thinnest at the distobuccal root of right the second molar (1.52 ± 0.85 mm). Ramanauskaite et al .[24] also measured the thickness of the maxillary sinus membrane and found that tooth vitality, residual alveolar bone height, and periodontal bone loss did not influence the thickness of the maxillary sinus membrane. Different from our study, the thickness of the membrane in each apical location was not shown in the study of Ramanauskaite et a l[24] Here we find that the thickness of membrane in each root apex differs significantly among roots from different people. Therefore, the thickness of membrane should be taken into account when assessing the risk before the treatment.

In the current study, the angle between the buccal and palatal roots is maximum in the right first molar of Type I (56.47 ± 29.30), and minimum in the left second molar of Type V (31.55 ± 26.03). Type I had larger angle than the other types (Type II-V). To our knowledge, this finding has not been published before.

Kwak et al .[12] and Tian et al .[16] analyzed the morphological characteristics of the maxillary sinus in Korean and Chinese patients and reported that the shortest distances were observed at the maxillary second molar (Table 7). However, the sample size used in the study conducted by Kwak et al .[12] was only 33, and Tian et al .[16] did not provide a detailed classification of the relationship between the roots and maxillary sinuses of the maxillary molars.

Experimental results have indicated that the anatomical relationship between the maxillary molars and the MSF is complex and impacts clinical operation s[4]. If clinicians do not fully understand the anatomical relationship between the affected teeth and the maxillary sinus through CBCT imaging, complications, such as maxillary sinus perforation or maxillary sinus infection, may occur during maxillary molar extraction s[25]. CBCT examination can estimate the risk of immediate implant surgery and help reduce the risk of failur e[26]. Understanding the relationship between the root tip and the maxillary sinus mucosa can prevent odontogenic maxillary sinusitis caused by root canal therap y[27].

In this study, the relationship between root apex of each tooth position and MSF definitely help clinicians to predict the risk of maxillary sinus fistula in the treatments of dental implant or root cannel therapy. Due to the significant individual variations, the CBCT scanning is recommended to assess the individual risk. However, based on the concept of ALARA (as low as reasonably achievable), the CBCT scanning only should be necessary when the operation is very close to the MSF.

## Conclusions

Among the root apices of the maxillary molars, the mesiobuccal root apex of the left second molar is closest to the MSF, and it has highest incidence of protrusion into the sinus. The unique anatomic relationships between the maxillary molars and the maxillary sinus in Chinese population allows dentists to provide adequate treatments; estimate the risk of maxillary posterior tooth extractions, dental implantations, and root canals; and reduce the incidence of maxillary sinusitis and other complications.

## Supplementary information

**Additional file 1.** Raw data of the measurement.

## Data Availability

The raw data can be found in Additional file 1: raw data to BMC oral health.

## Abbreviations

CBCT: Cone-Beam Computed Tomography
CTPR: The cortical thickness of the MSF closest to the palatal root apex;
DDBR: The distance between the distobuccal root apex and the inferior wall of the MSF
DMBR: The distance between the mesiobuccal root apex and the inferior wall of the MSF
DPR: The distance between the palatal root apex and the inferior wall of the MSF CTMBR The cortical thickness of the MSF closest to the mesiobuccal root apex CTDBR The cortical thickness of the MSF closest to the distobuccal root apex
MSF: Maxillary sinus floor
MTMBR: The mucosa thickness of the MSF closest to the mesiobuccal root apex MTDBR The mucosa thickness of the MSF closest to the distobuccal root apex
MTPR: The mucosa thickness of the MSF closest to the palatal root apex
